# Endoscopic Management of Jejunal Perforation During Endoscopic Ultrasonography: A Case Report and Literature Review

**DOI:** 10.7759/cureus.43265

**Published:** 2023-08-10

**Authors:** Ngoda Manongi, Steven Tsistrakis

**Affiliations:** 1 Internal Medicine, NewYork-Presbyterian Queens, Flushing, USA; 2 Gastroenterology, Androscoggin Valley Hospital, Berlin, USA

**Keywords:** iatrogenic, intestinal perforation, over-the-scope clips, jejunum, endoscopic ultrasonography

## Abstract

Endoscopic ultrasound is a useful diagnostic and interventional device for gastroenterologists. Although extremely useful, endoscopic ultrasound is not a benign tool. Possible complications of endoscopic ultrasound include hemorrhage, infection, and perforation. Although rare, iatrogenic small bowel perforations have been reported largely on the duodenum and rarely on the jejunum or ileum. Traditionally, these iatrogenic small bowel perforations have been managed with open surgery. However, recent emerging clinical data has revealed that immediate endoscopic treatment may be a feasible and safe alternative to surgery in select cases. Here, we describe the endoscopic management of an iatrogenic jejunal perforation during a linear endoscopic ultrasound examination managed successfully using an endoscopic clip.

## Introduction

Endoscopic ultrasound (EUS) has been a crucial tool for gastroenterologists since its early days in the 1980s. Although extremely useful as a diagnostic and interventional tool, EUS is not a benign tool. Possible complications of EUS include hemorrhage, infection, and perforation [1,2]. The first reported endoscopic closure in a patient with gastrointestinal perforation was reported at the end of the 1990s [3]. Endoscopic closure, especially using endoclips, has the advantages of being effective, being minimally invasive, and having a shorter recovery time, which makes it an ideal treatment modality for gastrointestinal perforation, especially duodenal perforation [4]. Currently, only a few large-scale studies have been conducted to verify the clinical significance of this technique [5]. Traditionally, surgery has been the therapeutic choice for the management of gastrointestinal perforations [6]. However, recently, data have emerged revealing that immediate endoscopic treatment with an endoclipping device may be an acceptable alternative to surgery in select cases [6-10]. In this report, we present successful endoscopic management with a clipping device of iatrogenic jejunal perforation that occurred during a linear EUS examination to demonstrate the feasibility of managing iatrogenic EUS-related jejunal perforations using endoscopic clipping devices.

## Case presentation

**Figure 1:**
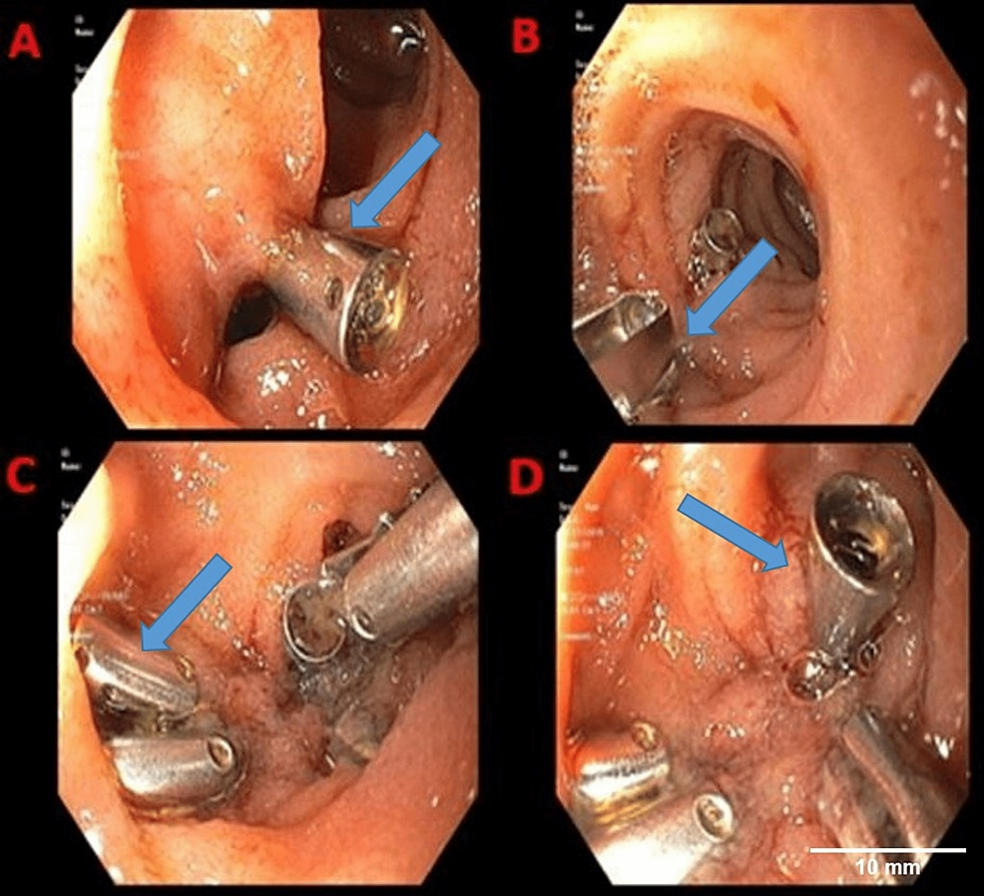
Multiple deployments of endoclips (blue arrowheads) at the afferent limb of gastrojejunal anastomosis.

A male patient in his late 60s with peptic ulcer disease status post-antrectomy with Billroth II reconstruction and myasthenia gravis presented with generalized weakness and multiple episodes of melena for three days. In the emergency room, the patient was hypotensive, and blood work revealed hemoglobin of 8.2 g/dL (normal >13 g/dL). Additionally, a computed tomography (CT) angiogram of the abdomen and pelvis with contrast revealed no evidence of active gastrointestinal hemorrhage; however, it showed pancreatic head-enhancing hypodense lesions, with concerns for neoplasm. A CT scan of the pancreas with contrast showed subcentimeter cysts in the region of the head of the pancreas without abnormal internal enhancement. Esophagogastroduodenoscopy revealed scattered mild inflammation characterized by edema and erythema in the entire examined stomach. In addition, evidence of a patent Billroth II gastrojejunostomy was found. The gastrojejunal anastomosis was characterized by ulceration. A 10 mm non-bleeding superficial gastric ulcer with a clean ulcer base (Forrest Class III) was found at the anastomosis. On the subsequent upper EUS, immediately after visualizing the liver, a *pop *sensation was felt as the EUS scope was being traversed through the afferent limb of Billroth II anastomosis. The scope was immediately withdrawn. The upper endoscope was re-inserted for evaluation. A perforation was found in the afferent limb 5-10 cm from the gastrojejunal anastomosis. This defect measured 10 mm × 15 mm in diameter. To repair the defect, the tissue edges were approximated and 10 hemostatic clips were successfully placed with the successful closure of the defect (Figure 1). There was no bleeding during or at the end of the procedure. The patient remained hemodynamically stable throughout the procedure. The patient was monitored in the surgical intensive care unit for three days. A repeat CT scan of the abdomen/pelvis revealed no free air. The diet was restarted on day three, and the patient was discharged back to the nursing home on day seven. A follow-up at two months in the outpatient clinic revealed patient doing well and at baseline.

## Discussion

The use and indications of EUS in the gastroenterology setting, including identification, staging, sampling, and follow-up of benign and malignant lesions, have become widespread. The ubiquity of EUS partly has been due to its low rate (2%) of complications [11]. Iatrogenic perforation remains one of the most common complications in EUS but is still very rare. The reported incidence of perforation during diagnostic endoscopy is less than 1% while that during therapeutic endoscopy is close to 6% [12]. The esophagus and duodenum involve most of the cases and very rarely the jejunum [13].

Perforation is a serious complication with a high mortality rate from dangerous medical conditions including infection of the peritoneum and septic shock. Iatrogenic perforations that occur during endoscopic procedures have traditionally been managed surgically [14]. However, with the rapid development of endoscopic devices and techniques, endoscopic management of perforations is transforming the way complications are managed [15]. Several reports of successful clip closures of gastrointestinal perforations resulting from a wide variety of causes have been published [16-18]. In fact, in a study of 121 gastric endoscopic mucosal resection perforations, 117 patients underwent endoscopic clip closure, with successful closure in 115 patients [17].

However, most of these reported cases have been endoscopic management of defects in the esophagus, stomach, duodenum, and colon [8,18]. Very few cases have been reported demonstrating the efficacy of endoclipping in jejunal perforations [19,20]. Therefore, our report showcases and demonstrates one of the few cases in which the jejunal was involved, widening the reach of endoscopic management of perforations in an area that once was solely a surgical arena. Moreover, this case demonstrates that endoscopic clipping devices in the hands of a well-trained operator are not only safe but also reliable. Additionally, the endoscopic clipping approach has been shown not to compromise the healing of the closed defect margins or cause clip impaction, perforation, or other significant complications [13].

Our case demonstrates that it is feasible to manage iatrogenic EUS-related jejunal perforations using endoscopic clipping devices. In select patients, especially depending on the size and location of the perforation, surgery can be avoided by using endoscopic clipping devices to repair the defects. It is imperative to be cognizant that endoscopic clipping success depends on various other factors, including the type of perforation, time of diagnosis, location, size, type of perforation, equipment availability, location and size, as well as the immediate recognition and closure of the defect.

## Conclusions

In this case, we demonstrated the safety, feasibility, and effectiveness of endoscopic closure by endoclips in a patient with iatrogenic EUS-related jejunum perforation. Potentially, using endoclips has a much lower rate of complications compared to open surgical intervention traditionally employed in these circumstances. However, the findings of this study need to be further validated by large-scale multicenter clinical trials due to the limitation of enrolling a small sample from a single center.
